# Bimodal Interphase Architecture in Filled Elastomers: Molecular Dynamics Evidence and Experimental Signatures

**DOI:** 10.3390/molecules31101615

**Published:** 2026-05-11

**Authors:** Yancai Sun, Haoran Wang, Peiwu Hou, Wenjuan Bai, Dianming Chu, Wenzhong Deng

**Affiliations:** 1 College of Mechanical and Electrical Engineering, Guilin University of Aerospace Technology, Guilin 541004, China; 2024025@guat.edu.cn; 2 College of Mechanical and Electrical Engineering, Qingdao University of Science and Technology, Qingdao 266061, China; 4025037014@mails.qust.edu.cn (P.H.); bwj@qust.edu.cn (W.B.); chudianming@qust.edu.cn (D.C.); 3 University Engineering Research Center of Non-Standard Intelligent Equipment and Process Control Technology, Guilin 541004, China; 4 Shandong Province Key Laboratory of Rubber-Based High-Performance Composites and Advanced Manufacturing, Qingdao 266061, China; 5 Guangxi Engineering Research Center for Advanced Process Manufacturing of Precious Metal New Materials, Guilin 541004, China; 6 China Chemical Sixth Construction Design and Research Institute, Wuhan 430074, China; whr150011@scccnce.com.cn

**Keywords:** bound rubber, interphase heterogeneity, molecular dynamics, filled elastomers, viscoelastic reinforcement

## Abstract

The polymer–filler interphase in filled elastomers is often represented by a single thickness, obscuring internal heterogeneity. Coupling coarse-grained molecular dynamics with dynamic mechanical analysis of EPDM/carbon-black compounds, we resolve a bimodal bound-rubber layer with a dense inner zone set by surface adsorption and a looser outer zone sustained by chain connectivity. Heating contracts the outer zone about twice as strongly as the inner zone (outer: 26.5%, 95% confidence interval 17.4–34.8%; inner: 13.3%). Per-layer mean-squared displacement analysis shows a modest mobility gradient between the 1–2 nm outer zone and the bulk. Dynamic mechanical analysis at 120–140 °C shows a flatter reinforcement factor at higher temperature, consistent with interphase-linked thermal contraction. Lengthening the chain at fixed filler loading markedly enlarges the bridging fraction and the cumulative excess thickness, signaling a transition from adsorption-limited to connectivity-limited reinforcement. These results show that a single interphase boundary can miss a dynamically active outer zone relevant to reinforcement and thermal aging in filled elastomers.

## 1. Introduction

Carbon black (CB) and silica reinforcement can raise the storage modulus of a rubber matrix by an order of magnitude, yet the molecular origin of the enhancement has remained incompletely resolved for more than half a century [1,2,3,4]. Classical mean-field theories such as the Guth–Gold equation treat the filler as a rigid inclusion that amplifies the strain field in the surrounding matrix, but do not account for the polymer–filler interphase—a region of altered chain packing and mobility extending several nanometers from the particle surface [5,6,7].

Experimental evidence from solid-state NMR [8], neutron scattering, and dielectric spectroscopy consistently points to the existence of a “bound rubber” layer whose chains exhibit reduced mobility compared with the bulk [9,10,11,12]. Litvinov et al. measured distinct NMR relaxation components in vulcanized EPDM/CB compounds and attributed the immobile fraction to chains adsorbed within 1–3 nm of the CB surface [8]. Berriot et al. confirmed a glassy shell around silica nanoparticles in model poly(ethyl acrylate) nanocomposites using differential scanning calorimetry and dynamic mechanical analysis [13]. Molecular dynamics (MD) simulations by Starr et al. showed that polymer segments near a nanoscopic particle experience cage-like confinement with subdiffusive mean-square displacement (MSD) [14], and Eslami et al. quantified interphase thickness using atomistic MD of silica in PMMA oligomers [15].

Complementary work has examined how the interphase responds to filler loading, particle size, and surface chemistry. Robertson et al. showed that the viscoelastic glass transition shifts by several degrees when particle size drops below 50 nm, implying that the interphase volume becomes a significant fraction of the matrix [16]. Kawak et al. demonstrated through coarse-grained simulations that nonlinear reinforcement arises from cooperative deformation of filler–polymer interphase networks, not from hydrodynamic amplification alone [17,18]. With calorimetric evidence for gradient-like $T_{g}$ profiles near filler surfaces [13], these studies indicate that the interphase is not a simple rigid shell but a spatially graded region whose properties depend on position, temperature, and deformation rate.

Despite this progress, a fundamental question persists: does the interphase possess resolvable internal structure, and if so, how does it couple to the macroscopic frequency-dependent reinforcement? Most cross-scale modeling efforts—including our companion study on EPDM/CB [19]—represent the interphase by a single scalar thickness $h_{\mathrm{bound}}$ fed into continuum constitutive models (e.g., the fractional Maxwell or Phan–Thien–Tanner model). While effective for calibrating macroscopic rheology, this approach obscures spatial variation within the bound layer.

We address this gap by combining coarse-grained MD simulations of a bead-spring model [20,21,22] with DMA characterization of EPDM/CB compounds. The simulations resolve the internal architecture of the polymer–filler interphase; the DMA data anchor the frequency-dependent reinforcement trends to a measurable observable. The specific deliverables addressed by these two channels are introduced at the start of Section 3.1, where the stratification, mobility-gradient and lever-tuning analyses begin.

## 2. Materials and Methods

### 2.1. Coarse-Grained Molecular Dynamics

All MD simulations employed a coarse-grained bead-spring model [20,23] implemented in LAMMPS (version 22 July 2025) [24,25]. Polymer chains of N=25, 50, or 100 beads were placed in a periodic cubic box with frozen spherical filler particles (type 2, $\sigma_{f}=3.0\;\sigma$). The Lennard–Jones (LJ) potential with $\varepsilon =1.0\;k_{B}T$, σ=1.0, and $r_{\mathrm{cut}}=2.5\;\sigma$ governed non-bonded interactions; bonded beads were connected by harmonic springs ($k=500\;\varepsilon /\sigma^{2}$, $r_{0}=0.97\;\sigma$). Throughout this work the LJ length unit is mapped to nanometers via σ≈0.5 nm, a conventional Kremer–Grest choice for EPDM-like chains in which each bead represents 5–10 backbone monomers; all nanometer values in the Results ($h_{90\%}$, $h_{99\%}$, $h_{\mathrm{excess}}$, $h_{\mathrm{bound}}$, bin widths) use this mapping. The approximate nature of the conversion is revisited in the Limitations.

The simulation protocol comprised five phases: (i) soft push-off with a ramped soft potential (0 → 300 over 60,000 steps at Δt=0.001τ); (ii) switch to the full LJ potential with brief NVE/limit equilibration; (iii) NPT density correction (500,000 steps); (iv) NVT equilibration (chain-length dependent: $2.5\times 10^{6}$ steps for N=25, $5\times 10^{6}$ for N=50, $10^{7}$ for N=100); and (v) NVT production ($10^{7}$ steps, Δt=0.005τ). The filler volume fraction ϕ was varied from 5% to 40% by adjusting the number of filler particles in the box. The temperature comparison discussed below uses flat-wall reference profiles at $T^{*}=0.6$, 0.9, 1.1, and 1.5 (three independent replicas each); separate $T^{*}=1.0$ nanocomposite series were used for the chain-length and filler-loading comparisons.

#### Wall-Potential Convention

**Figure 1:**
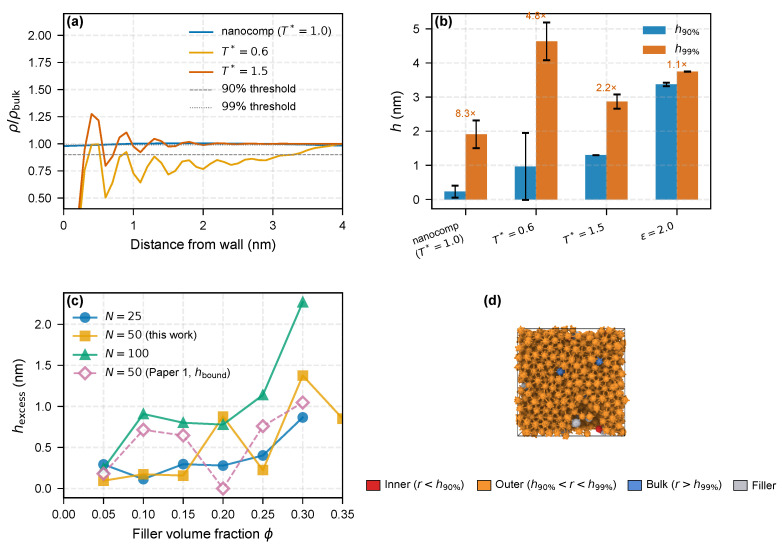
Bimodal interphase stratification. (**a**) Normalized flat-wall density profiles: *nanocomp* reference (12–6 LJ, $\varepsilon_{\mathrm{wall}}=1.0$, $T^{*}=1.0$) and $T^{*}=0.6$, 1.5 traces from the 9–3 wall setup ($\varepsilon_{\mathrm{wall}}=2.0$); dashed and dotted lines mark 90% and 99% of bulk density. (**b**) Grouped $h_{90\%}$ and $h_{99\%}$ bars with annotated $h_{99\%}/h_{90\%}$; bars from different setups are not directly comparable (Table 1). (**c**) $h_{\mathrm{excess}}$ versus ϕ for N=25, 50, 100 (this work, filled) with companion-study N=50 $h_{\mathrm{bound}}$ ([19], open diamonds). (**d**) Rendered nanocomposite cell (N=50, ϕ=10%, $T^{*}=1.0$): beads colored by distance to filler surface—red ($r<h_{90\%}$), orange ($h_{90\%}<r<h_{99\%}$), blue (bulk); thresholds use the flat-wall *nanocomp* reference (**b**). Direct radial quantiles for this geometry are in Figure S2.

**Figure 2:**
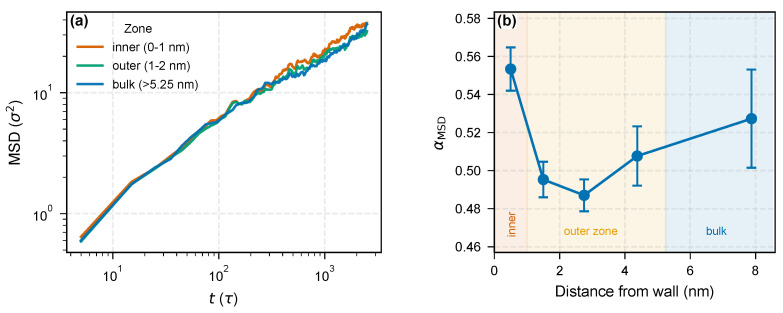
Spatially resolved chain dynamics from per-layer MSD analysis (flat wall, $T^{*}=1.1$, $\varepsilon_{\mathrm{wall}}=2.0$). (**a**) MSD(*t*) for three representative layers—inner (0–1 nm, red), outer (1–2 nm, green), and bulk (>5.25 nm, blue). (**b**) Per-layer subdiffusive exponent $\alpha_{\mathrm{MSD},\mathrm{layer}}$ versus distance from the wall; error bars are block-bootstrap σ of the log–log slope over 100≤t≤2500τ (block size 25, B=2000; see Section 2 and Supplementary Materials Table S3).

**Figure 3:**
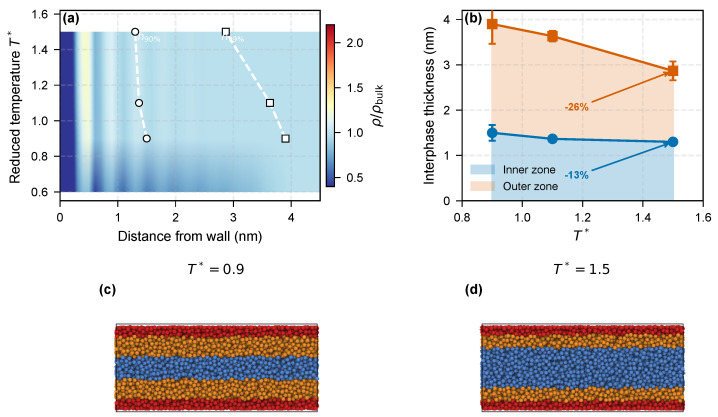
Thermal selectivity of the bimodal interphase. (**a**) Density-profile heatmap $\rho /\rho_{\mathrm{bulk}}$ versus distance from wall and reduced temperature (representative replica), with white markers tracing the three-replica mean $h_{90\%}$ and $h_{99\%}$ over $T^{*}=0.9$–1.5. (**b**) Inner ($h_{90\%}$) and outer ($h_{99\%}$) boundaries versus temperature (mean ± std, 3 replicas); shaded bands mark the two zones. Outer zone contracts 26.5% from $T^{*}=0.9$ to 1.5 (95% CI [17.4,34.8]%, d=3.03); inner zone only 13.3% (d=1.63); full statistics in Table S1. (**c**,**d**) Side-view snapshots of the flat-wall film at $T^{*}=0.9$ (broad outer band) and $T^{*}=1.5$ (outer band contracts); beads colored as in Figure 1d.

**Figure 4:**
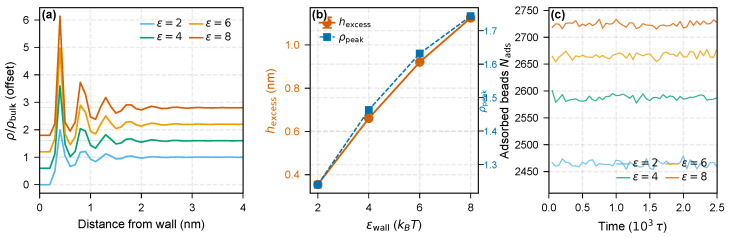
Wall interaction sensitivity ($\varepsilon_{\mathrm{wall}}=2$–8). (**a**) Stacked density profiles with uncertainty bands from three independent replicas. Stronger attraction produces a denser, wider adsorbed layer. (**b**) Cumulative excess integral $h_{\mathrm{excess}}$ (red) and peak normalized density (blue) versus $\varepsilon_{\mathrm{wall}}$, showing monotonic growth with error bars. (**c**) Adsorbed-bead count $N_{\mathrm{ads}}$ time series during the production run, summed over the two walls. $N_{\mathrm{ads}}$ is the LAMMPS count() of the dynamic group of polymer beads inside a 3σ-thick near-wall slab at each wall (*beads*, not chains); the quantity is used only as a time-averaged densification proxy for the adsorbed layer.

Three flat-wall setups are used and should not be conflated (Table 1). (i) The confined-melt reference *nanocomp* uses a 12–6 Lennard–Jones polymer–wall interaction with $\varepsilon_{\mathrm{wall}}=1.0\;k_{B}T$ (polymer–wall interaction energy parameter; identical to the bulk polymer–polymer parameter), so the reference film isolates the geometric contribution of confinement as the baseline for bimodal stratification analysis; it contributes only the *nanocomp* traces in Figure 1a,b. (ii) The temperature series ($T^{*}=0.6$–1.5) uses a Steele-type 9–3 wall potential (LAMMPS wall/lj93) with $\varepsilon_{\mathrm{wall}}$ fixed at $2.0\;k_{B}T$; this setup underlies the temperature-dependent $h_{90\%}/h_{99\%}$ traces in Figure 1a,b, the per-layer MSD analysis in Figure 2 (run at $T^{*}=1.1$), and the thermal-selectivity analysis in Figure 3. (iii) A surface-chemistry sensitivity study uses the same 9–3 potential with $\varepsilon_{\mathrm{wall}}$ varied from 2 to $8\;k_{B}T$ (three independent replicas each); this setup provides the ε=2.0 bar in Figure 1b and Figure 4. Because the three setups differ in wall-potential form and/or interaction strength, their interphase thicknesses are not directly comparable; cross-setup comparisons are qualitative only (Section 3.5).

**Table 1:** Flat-wall setups used in this work. The three setups differ in wall-potential form and interaction strength, so $h_{90\%}$ and $h_{99\%}$ values are not quantitatively comparable across setups. Assignments are resolved at the panel level because Figure 1a,b overlay traces from more than one setup.

Use Case	Potential	$\varepsilon_{\mathrm{wall}}\;(k_{B}T)$	Figure Panels
Bimodal reference (*nanocomp*)	12–6 LJ	1.0	Figure 1a,b nanocomp traces only
Temperature series	9–3 (wall/lj93)	2.0	Figure 1a,b $T^{*}=0.6$/1.5 traces, Figure 2 and Figure 3
Surface-chemistry sensitivity	9–3 (wall/lj93)	2.0–8.0 (swept)	Figure 1b ε=2.0 bar, Figure 4

*Note:* Figure 1c,d report direct nanocomposite simulations with spherical fillers (not flat-wall) and are therefore listed separately in Section 3.1.

During production, three output streams were recorded: (a) chunk-averaged density profiles (production bin width 0.5σ, sampled every $10^{5}$ steps, used for the global overview in Figure 1a); (b) center-of-mass MSD of the polymer group (sampled every $10^{3}$ steps); (c) stress autocorrelation for Green–Kubo analysis (correlator length $2\times 10^{4}$ steps). For the flat-wall density-threshold analysis in Section 2.2 the same trajectories are re-binned at a finer 0.2σ (=0.1 nm) resolution to resolve the $h_{90\%}/h_{99\%}$ crossings; for the nanocomposite cumulative-excess analysis the native 0.5σ output is used directly. All three bin widths refer to the *same* underlying trajectory data, only differing in the post-processing resolution used.

**Figure 5:**
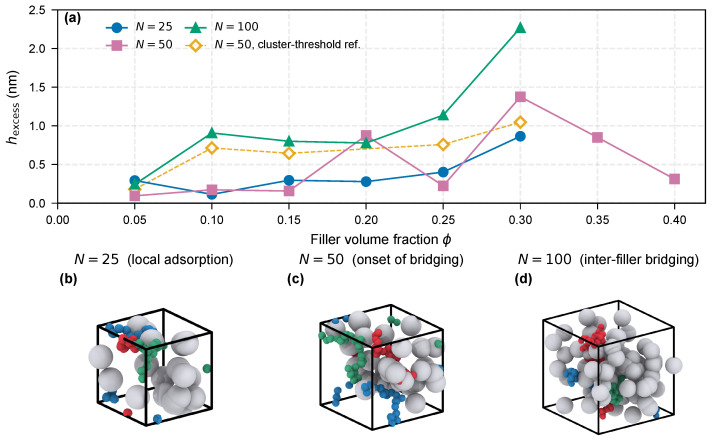
Chain length effect on interphase size and topology ($h_{\mathrm{excess}}$ is a cumulative excess integral in length units, not a geometric layer thickness). (**a**) $h_{\mathrm{excess}}$ versus ϕ for N=25, 50, 100 (this work, filled) and companion-study N=50$h_{\mathrm{bound}}$ ([19], open diamonds); the quantitative claim is the across-*N* contrast at fixed loading (see text for within-*N* scatter). (**b**–**d**) Whole-box renderings at ϕ=10% for N=25, 50, 100: fillers silver, the three longest chains highlighted (crimson, emerald, steel blue); ensemble bridging statistic in Figure S4.

The MSD power-law exponent is extracted from a linear fit to log[MSD(t)] versus logt over the latter half of each production trajectory (Δt=0.005τ). Two fitting protocols are labeled throughout: (i) the *per-layer* protocol $\alpha_{\mathrm{MSD},\mathrm{layer}}$, applied to the flat-wall geometry (Figure 2), in which polymer beads are binned into five static layers by their initial distance from the nearest wall and the exponent is fitted layer-by-layer; and (ii) the *ensemble* protocol $\alpha_{\mathrm{MSD},\mathrm{ens}}$, applied to the nanocomposite loading series (Figure 5 and Table 2), in which a single exponent is fitted to the aggregated MSD of all polymer beads. The two protocols probe different physics—spatial mobility gradient within one film versus loading-averaged mobility across nanocomposite morphologies—and their numerical values are not comparable. Because the quality of the log–log fit is not uniform across all loading points, $\alpha_{\mathrm{MSD},\mathrm{ens}}$ is used below as a secondary mobility indicator. Uncertainty on each per-layer exponent (Figure 2b) is estimated by a moving-block bootstrap (B=2000 resamples, 25-point contiguous blocks) on the (logt,logMSD) samples over 100≤t≤2500τ; block resampling preserves the serial correlation of the MSD trace and is robust to the block-size choice (Table S3). Full simulation details are listed in the Supplementary Materials PDF; the underlying LAMMPS input scripts, Python 3.10 analysis and figure-generation code, and processed CSV data files are available as stated in the Data Availability Statement.

**Table 2:** Chain length effect on the cumulative excess integral $h_{\mathrm{excess}}$ at ϕ=10%, $T^{*}=1.0$. All three rows use the same excess-integral metric $h_{\mathrm{excess}}$ (reported in length units but not equivalent to a geometric layer thickness) and the same ensemble log–log MSD-fitting protocol ($\alpha_{\mathrm{MSD},\mathrm{ens}}$), so the entries are directly comparable. The N=50 exponent is fitted over a narrower well-behaved window than the N=25 and N=100 traces (noisier log–log slopes at short chains); the non-monotonic ordering in the $\alpha_{\mathrm{MSD},\mathrm{ens}}$ column therefore reflects fit-window sensitivity rather than a physical inversion, and $h_{\mathrm{excess}}$ is the primary interphase-size metric. Values are persisted to data/md_hbound/chain_length_scaling_table2.csv, derived from the corresponding simulation summary tables and retained as a reproducible data artefact.

*N*	$h_{\mathrm{excess}}$ (nm)	$\alpha_{\mathrm{MSD},\mathrm{ens}}$	Interphase Regime
25	0.11	0.18	Adsorption-dominated
50	0.17	0.56	Crossover (onset of bridging)
100	0.91	0.34	Connectivity-assisted

### 2.2. Multi-Percentile Interphase Analysis

Two complementary metrics were used, each suited to its respective geometry.

*Flat-wall systems (density-threshold method)*. For flat-wall simulations, the normalized density profile $\rho \left(z\right)/\rho_{\mathrm{bulk}}$ was computed with a bin width of 0.2σ (=0.1 nm), where $\rho_{\mathrm{bulk}}$ is the median density in the central 20% of the simulation box. The *p*-th percentile interphase thickness $h_{p}$ is the smallest distance *z* from the wall at which the smoothed density profile first recovers to the fraction *p* of the bulk value: $\rho \left(h_{p}\right)/\rho_{\mathrm{bulk}}\geq p$. Thus $h_{90\%}$ marks the distance at which the density has recovered to 90% of $\rho_{\mathrm{bulk}}$ (inner zone boundary), while $h_{99\%}$ marks the more distant point at which it reaches 99% (outer zone boundary). The stratification ratio $h_{99\%}/h_{90\%}$ quantifies the degree of structural heterogeneity within the interphase.

*Structural rendering*. Representative 3D configurations in Figure 1d, Figure 3c,d and Figure 5b were rendered from LAMMPS final.data snapshots with OVITO 3.12 using the Tachyon ray-tracer (ambient occlusion and shadows enabled). For bimodal stratification panels, polymer beads were colored by distance to the nearest filler (nanocomposite) or wall (flat-wall), using the measured $h_{90\%}$ and $h_{99\%}$ as band boundaries. For chain-topology panels, the simulation cell was drawn in a thick black outline, filler beads were kept silver and opaque, and every polymer chain except the three with the largest periodic-image-corrected spatial extent was deleted. The three retained chains were rendered in crimson, emerald and steel blue. Identical orthographic viewports (zoom_all) were used for N=25, 50 and 100 so the three panels are directly comparable. Harmonic bonds were rendered as cylinders from the particle connectivity of the retained chains (consistent with the harmonic bond potential, $k=500\;\varepsilon /\sigma^{2}$, $r_{0}=0.97\;\sigma$, used throughout the simulations).

*Nanocomposite systems (cumulative excess integral)*. For nanocomposite simulations with distributed spherical fillers, the geometry-dependent positioning of filler surfaces makes a single distance coordinate ill-defined. Instead, we characterize the adsorbed layer by the cumulative excess-density integral (Equation (1)),

(1) $$h_{\mathrm{excess}}\;=\;\int_{0}^{\infty }\max\;\left[\;\frac{\rho (r)}{\rho_{\mathrm{bulk}}}-1,\;0\right]dr,$$

where *r* is the distance from the nearest filler surface (bin width 0.5σ). This scalar collapses the full excess-density profile into a single, geometry-independent measure of total adsorbed material, directly comparable across different filler volume fractions ϕ.

### 2.3. Dynamic Mechanical Analysis

DMA experiments were performed on a TA Instruments Q800 analyzer (TA Instruments, New Castle, DE, USA) in shear sandwich mode. Four uncured industrial EPDM compounds were tested: three filled compounds labeled by target post-cure Shore A hardness (EPDM60, EPDM70, EPDM80; fixed carbon-black loading, paraffinic oil as the hardness-controlling variable) and an unfilled EPDM carrying the same cure package as the Shore 60 grade as matrix reference. Shore A labels follow industrial nomenclature for the post-cure hardness.

Paired rectangular specimens (10.0 × 8.0 × 2.0 mm each, cold-cut from compounded sheets) were mounted on both sides of the shear-sandwich central tongue with a 0.5 N static compressive pre-load to ensure gap-free contact; all measurements were conducted under high-purity N_2_ at 50 mL/min to suppress thermal-oxidative aging during the high-temperature sweeps. Prior to the first sweep on each specimen, a strain-amplitude scan ($\gamma_{0}=0.01$–2%, 1 Hz, 120 °C) was used to locate the linear viscoelastic region (LVR); the LVR limit $\gamma_{c}$ was taken at the 5% drop of $G^{\prime }$ from its small-strain plateau, yielding $\gamma_{c}=0.42\pm 0.08$% for the stiffest grade (EPDM80). All frequency sweeps used $\gamma_{0}=0.1$%, well within the LVR for every sample. Each specimen was equilibrated at the target temperature for 15 min before each sweep, and three independent specimens per formulation were tested; reported moduli are specimen-mean ± standard deviation. The 120–140 °C measurement window is below the cure-package activation threshold: each specimen was measured at 120 °C and 140 °C before being raised to 160 °C, where cure activation first became evident ($G^{\prime }$ reversal and tanδ drop below 0.5; Section 3.3); the N_2_ atmosphere additionally suppresses oxidative crosslinking. No formal time-sweep test was performed.

Isothermal frequency sweeps (0.01–100 Hz, 41 logarithmically spaced points, 10 per decade) were conducted at 120, 140, and 160 °C. The 160 °C isotherm is treated as a post-cured reference state: for the Shore 60 and Shore 70 grades $G^{\prime }\left(\omega \right)$ increases from 140 to 160 °C and tanδ falls below 0.5, inconsistent with ordinary thermal softening and diagnostic of cure-package activation during the high-temperature ramp. The frequency-slope analysis of Section 3.3 therefore uses only the 120 and 140 °C isotherms; the 160 °C point is shown as an open symbol but not interpreted as equilibrium. The reinforcement factor was computed as $R\left(\omega \right)=G_{\mathrm{filled}}^{\prime }\left(\omega \right)/G_{\mathrm{pure}}^{\prime }\left(\omega \right)$, where $G_{\mathrm{pure}}^{\prime }$ is the same-batch unfilled EPDM storage modulus measured under identical conditions, avoiding literature-sourced reference spectra. Uncertainties in R(ω) were propagated from the triplicate-specimen standard deviations of numerator and denominator via $\sigma_{R}/R=\sqrt{{\left(\sigma_{G_{f}^{\prime }}/G_{f}^{\prime }\right)}^{2}+{\left(\sigma_{G_{p}^{\prime }}/G_{p}^{\prime }\right)}^{2}}$, combining three independent sources of random error: (i) specimen-to-specimen variation (cold-cut geometry, batch inhomogeneity), (ii) torque resolution at the lowest frequencies (ω≤0.1 rad/s, where signal-to-noise is limiting), and (iii) instrument-level drift between the filled and matrix-reference measurements (minimized by same-day, same-calibration pairing). Systematic errors common to both numerator and denominator (temperature calibration, gap-setting error) cancel in R(ω) by construction. Hardness varies through plasticizer-controlled dilution (60, 40, and 20 phr paraffinic oil for EPDM60/70/80; Table 3) at fixed N550 loading (80 phr), so Shore A is the ordering index for R(ω). MD nanocomposite scans of ϕ are an independent mechanistic variable and do not map one-to-one onto the three experimental compounds.

**Table 3:** Four EPDM samples used for DMA; all samples were tested in the uncured state. The Shore A column reports the target post-cure hardness of the vulcanizate, used here as the industrial sample identifier. Carbon-black loading (N550, 80 phr) is fixed across the three filled compounds; the listed paraffinic oil phr is the primary hardness-controlling formulation variable. The Pure EPDM sample is the unfilled matrix reference (no CB, no paraffinic oil) carrying the same cure package as the Shore 60 grade and serves as the pure-matrix denominator for $R\left(\omega \right)=G_{\mathrm{filled}}^{\prime }/G_{\mathrm{pure}}^{\prime }$.

Sample	Role	Shore A	Oil (phr)
Pure EPDM	matrix reference	—	0
EPDM60	filled compound	60	60
EPDM70	filled compound	70	40
EPDM80	filled compound	80	20

## 3. Results

### 3.1. Bimodal Interphase Stratification

The Results section addresses three deliverables in sequence. First (this subsection and Section 3.2), we quantify the stratification of the bound layer through two complementary thickness metrics—the density-threshold pair ($h_{90\%}$, $h_{99\%}$) for flat-wall reference systems and the cumulative excess integral $h_{\mathrm{excess}}$ for nanocomposite loading series (Equation (1))—reporting two physically distinct measures (the inner/outer boundary pair and the total adsorbed excess). Second (Section 3.2), we resolve the spatial mobility gradient via two protocols, the per-layer exponent $\alpha_{\mathrm{MSD},\mathrm{layer}}$ for the flat-wall geometry and the ensemble exponent $\alpha_{\mathrm{MSD},\mathrm{ens}}$ for the nanocomposite loading series, and identify which zone boundary controls the dynamically suppressed volume. Third (Section 3.3 and Section 3.4), we examine how filler volume fraction ϕ, temperature $T^{*}$, wall interaction strength $\varepsilon_{\mathrm{wall}}$, and chain length *N* reshape the bimodal interphase, and whether the temperature-dependent structural contraction leaves a detectable signature in the experimental reinforcement factor R(ω).

Figure 1 presents the multi-percentile analysis of the polymer–filler interphase. For the confined-melt flat-wall reference system (nanocomp), the density profile recovers to 90% of the bulk value at $h_{90\%}=0.23$ nm, but does not reach the 99% threshold until $h_{99\%}=1.91$ nm, yielding a stratification ratio of 8.3. This indicates that a thin, densely adsorbed inner zone is surrounded by a much broader outer zone of weakly perturbed chains. The ε=2.0 system, which models a stronger polymer–filler attraction, shows $h_{90\%}=3.37$ nm and $h_{99\%}=3.75$ nm (ratio 1.1), consistent with a more uniform, strongly bound interphase.

For the nanocomposite ϕ-series (N=25), the excess density integral $h_{\mathrm{excess}}$ ranges from 0.29 nm at ϕ=5% to 0.87 nm at ϕ=30% (Figure 1c). The trend is non-monotonic, with the ϕ=10% point falling to 0.11 nm before recovering at higher loadings. As discussed in Section 3.4, this low value is interpreted within the adsorption-dominated regime of short chains, where individual filler surfaces decorate their local shells without establishing connectivity between fillers; the point-to-point scatter reflects the sensitivity of $h_{\mathrm{excess}}$ to the specific filler-packing realization at each loading. A power-law fit over the full N=25 dataset gives $h_{\mathrm{excess}}\propto \phi^{0.40}$ and is reported as an order-of-magnitude descriptor, not a mechanistic scaling law.

At the intermediate chain length N=50 a new ϕ-series uses the same excess-density-integral metric. Over ϕ=5–30%, $h_{\mathrm{excess}}$ spans 0.09–1.38 nm with strongly non-monotonic dependence, jumping to 0.88 nm already at ϕ=20%. For cross-method validation we overlay the cluster-threshold $h_{\mathrm{bound}}$ values reported previously [19], which range from 0.18 nm (ϕ=5%) to 1.05 nm (ϕ=30%) and follow a smoother power law $h_{\mathrm{bound}}\propto \phi^{0.84}$. The two metrics agree in order of magnitude; the broader scatter of $h_{\mathrm{excess}}$ reflects its stronger sensitivity to specific filler-packing realizations at this chain length.

The bimodal stratification reflects the competition between adsorption energy and conformational entropy. In the inner zone, strong van der Waals attraction ($\varepsilon_{\mathrm{wall}}=4$–8 $k_{B}T$) outweighs the entropy penalty of flattened conformations, producing a dense adsorbed layer ($\rho_{\mathrm{peak}}/\rho_{\mathrm{bulk}}\approx 3.0$–4.3 across the $\varepsilon_{\mathrm{wall}}=4$–8 $k_{B}T$ sweep; see Section 3.4 and Figure 4) whose thermal response is weaker than that of the outer zone (Section 3.2). In the outer zone, chains are tethered through backbone connectivity rather than direct surface contact; this region is sustained at the cost of conformational entropy and is therefore thermally labile—a prediction tested quantitatively in the next section.

As a consistency check on the nanocomposite geometry, we built a direct radial density profile $\rho \left(r\right)/\rho_{\mathrm{bulk}}$ in the N=50, ϕ=10, 20 and 30% systems, with *r* measured to the nearest filler surface and the shell volume estimated by Monte-Carlo sampling (to account for overlapping shells at high ϕ; Figure S2). The profile shows a clear enhancement of $\rho \left(r\right)/\rho_{\mathrm{bulk}}$ within the first 1–2 nm, returning to the matrix reference beyond, with finite and ordered cumulative-excess quantiles $h_{90\%}^{\mathrm{cum}}$/$h_{99\%}^{\mathrm{cum}}$ at all three loadings (Figure S2; operationally distinct from the flat-wall first-crossing $h_{90\%}$/$h_{99\%}$ used earlier in this section and in Figure 1b). The direct-nanocomposite stratification ratio $h_{99\%}^{\mathrm{cum}}/h_{90\%}^{\mathrm{cum}}$ is much smaller than the flat-wall first-crossing ratio $h_{99\%}/h_{90\%}$ (∼1.1 at ϕ=10% vs. 8.3 in the confined-melt reference), expected because at this filler density neighboring Voronoi cells overlap and suppress the low-density plateau; the nanocomp data are consistent with, but do not independently decompose, the flat-wall bimodal structure.

### 3.2. Thermal Selectivity of the Bimodal Interphase

The bimodal density structure has a dynamical counterpart. To quantify the mobility gradient directly, we divided the flat-wall system into five layers based on distance from the nearest wall and tracked the MSD of each layer independently over the production run (Figure 2). The per-layer subdiffusive exponent $\alpha_{\mathrm{MSD},\mathrm{layer}}$ [26], obtained from a block-bootstrap OLS fit on the log–log slope over the stable late-time window 100≤t≤2500τ (25-point contiguous blocks, B=2000 resamples; same definition used throughout this paper and in Supplementary Materials Table S3), is slightly lower in the 1–2 nm outer-zone layer ($\alpha_{\mathrm{MSD},\mathrm{layer}}=0.495\pm 0.009$) than in the bulk reference layer (z>5.25 nm, $\alpha_{\mathrm{MSD},\mathrm{layer}}=0.527\pm 0.026$), i.e., a modest reduction of Δα≈0.032 (∼6%). The per-zone bootstrap $\sigma_{\alpha }$ ranges from 0.009 to 0.026 across the five layers, and the large bulk error bar reaches into outer-zone values, so the outer-to-bulk contrast lies near the edge of within-window significance. We interpret this shift as consistent with a mobility stratification within the outer zone rather than definitive evidence on its own; the primary dynamical evidence for the bimodal architecture is the thermal-response contrast in Section 3.2. The innermost 0–1 nm layer registers a slightly higher apparent value ($\alpha_{\mathrm{MSD},\mathrm{layer}}\approx 0.55$); this is attributed to the static layer assignment mixing persistently adsorbed segments with chains that exchange with adjacent layers during the run, and should not be interpreted as a genuine mobility gradient within the inner zone. The flat-wall mobility contrast is small but directionally stable, and it is $h_{99\%}$—not $h_{90\%}$—that marks the outer boundary of the dynamically suppressed volume, consistent with the density-profile analysis of Section 3.1.

The inner and outer zones are governed by different energy scales—enthalpic adsorption versus entropic connectivity—and should therefore respond differently to heating. We tested this with flat-wall simulations at four temperatures ($T^{*}=0.6$, 0.9, 1.1, 1.5) using the 9–3 wall potential with $\varepsilon_{\mathrm{wall}}=2.0$ (the same setup as the ε=2.0 condition in Figure 1b, distinct from the $\varepsilon_{\mathrm{wall}}=1.0$ *nanocomp* reference). Results analyzed with the density-threshold method (bin width 0.2σ = 0.1 nm) are summarized in Figure 3 and Table 4.

The robustness of these numbers to the threshold choice *p* and to the density-profile binning is documented in the Supplementary Materials (Figure S1, Table S1): scanning *p* from 0.85 to 0.995 and comparing the raw 0.2σ bin, a 0.4σ rebin, and a 3-point smoothing alternative, the outer-zone contraction from $T^{*}=0.9$ to 1.5 remains positive in all cases, with an averaged magnitude of 19–28% across binning choices and an overall range of 13–37% across the seven thresholds; the reported 26% value (at p=0.99) lies near the middle of this distribution rather than at an extremum. Each temperature was simulated with three independent replicas (different random seeds) to quantify uncertainty. The outer-zone boundary $h_{99\%}$ contracts from 3.90 ± 0.44 nm to 2.87 ± 0.21 nm between $T^{*}=0.9$ and $T^{*}=1.5$, a 26.5% reduction (bootstrap 95% CI [17.4,34.8]%; Cohen’s d=3.03; exact permutation p=0.050). The inner-zone boundary $h_{90\%}$ contracts over the same window (1.50 ± 0.17 nm → 1.30 nm, 13.3% reduction, 95% CI [7.1,23.5]%, d=1.63, permutation p=0.050; the inter-replica standard deviation at $T^{*}=1.5$ falls below the 0.1 nm bin resolution and should be read as an upper bound of ≲0.05 nm rather than true zero variance), so the inner zone is not unchanged on heating but responds more weakly than the outer zone. With n=3+3 the permutation-test floor is p=1/20=0.050, so group-wise *p*-values alone cannot discriminate the two zones. A paired contrast $\Delta_{99}-\Delta_{90}=+13.2$ percentage points (paired bootstrap 95% CI [+7.5,+18.3], exact paired permutation p=0.050; Supplementary Table S1c) excludes zero at the 95% level, providing a second independent line of evidence that the outer zone responds more strongly than the inner zone. In effect-size terms $d_{99\%}$ is roughly twice $d_{90\%}$. This differential contraction is the central structural signature of the bimodal interphase: the inner adsorbed zone (adsorption energy $\gg k_{B}T$) responds only weakly to heating, whereas the outer zone—sustained by chain connectivity at a conformational-entropy cost—is progressively eroded by thermal activation. The $T^{*}=0.6$ system is anomalous (large replica-to-replica scatter, $h_{90\%}$ = 0.97 ± 0.98 nm), indicating that at this low temperature the polymer approaches vitrification and the sharp inner/outer distinction is replaced by a broad, frozen interphase.

Surface chemistry provides an orthogonal control. The interphase response to the polymer–filler interaction strength is shown in Figure 4. As $\varepsilon_{\mathrm{wall}}$ increases from 2 to 8 $k_{B}T$, the excess density integral $h_{\mathrm{excess}}$ grows monotonically from 0.35 to 1.12 nm, and the number of adsorbed polymer beads $N_{\mathrm{ads}}$ (those lying within 3σ of the wall, summed over the two walls of the thin film; LAMMPS count() of the dynamic near-wall atom group, not a chain count) increases from 2464 to 2725. The peak normalized density $\rho_{\mathrm{peak}}/\rho_{\mathrm{bulk}}$ rises from 1.24 to 1.74. These trends confirm that stronger filler–polymer attraction widens and densifies the bound layer, consistent with the enthalpic origin of the inner adsorbed zone [7,15]. Surface treatment or coupling agents that modify ε thus provide a lever for tuning the total adsorbed layer thickness and density, and by extension the overall interphase architecture.

**Figure 6:**
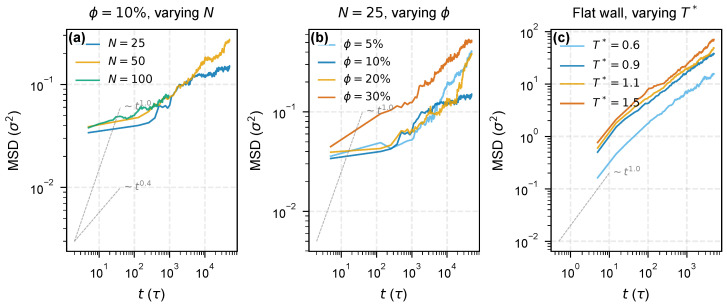
Mean-square displacement across three parameter axes. (**a**) Chain length effect: MSD(*t*) for N=25, 50, and 100 at ϕ=10%, showing clear chain-length-dependent separation, with the N=50 and 100 curves remaining above the N=25 curve over most of the sampled window. (**b**) Filler loading effect: MSD(*t*) for N=25 at four loadings, showing strong loading-dependent changes in both amplitude and slope without a simple monotonic ordering in the raw MSD magnitude. (**c**) Temperature effect: flat-wall MSD(*t*) at four temperatures, confirming thermal activation of chain mobility. Dashed lines indicate reference scaling exponents.

The selective outer-zone contraction matches the gradient $T_{g}$ picture of Berriot et al. [13], in which segments farther from the surface reach their glass transition at lower temperatures and desorb more readily upon heating. The heatmap in Figure 3a carries the same signal: the high-density band at z≈0.4 nm is preserved across $T^{*}=0.6$–1.5, while the broader z>1 nm shoulder fades progressively with rising temperature. The temperature dependence of the MSD across all three parameter axes (Figure 6c) confirms that thermal activation raises chain mobility without erasing the near-wall subdiffusion signature.

**Table 4:** Temperature dependence of interphase thickness metrics from flat-wall simulations ($\varepsilon_{\mathrm{wall}}=2.0$, N=50, density-threshold method, bin width =0.2σ). Values are mean ± std over three independent replicas with different random seeds.

$T^{*}$	$h_{90\%}$ (nm)	$h_{99\%}$ (nm)
0.6	0.97 ± 0.98 *^a^*	4.63 ± 0.55
0.9	1.50 ± 0.17	3.90 ± 0.44
1.1	1.37 ± 0.06	3.63 ± 0.12
1.5	1.30 ± < 0.05 *^b^*	2.87 ± 0.21

*^a^* Large scatter at $T^{*}=0.6$ reflects the onset of vitrification (some replicas frozen in broad disordered state). *^b^* The three replicas return the same $h_{90\%}$ crossing to within the 0.1σ=0.05 nm binning resolution of the density profile; the entry should be read as an upper bound of ≲0.05 nm on the replica-to-replica scatter, not as a true zero variance.

### 3.3. Experimental Signatures in Frequency-Dependent Reinforcement

**Figure 7:**
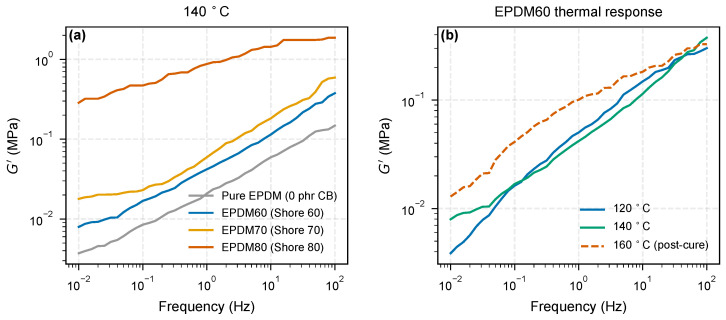
Dynamic mechanical analysis of EPDM/CB compounds. (**a**) Storage modulus $G^{\prime }\left(\omega \right)$ at 140 °C for the four samples: Pure EPDM (matrix reference), EPDM60, EPDM70, and EPDM80. The modulus rises monotonically with Shore A hardness and spans more than a decade between Pure EPDM and EPDM80. (**b**) Temperature evolution of $G^{\prime }$ for EPDM60 at three temperatures. The 120 → 140 °C step is classical thermal softening; the 160 °C curve (dashed) lies above 140 °C, indicating residual vulcanization during the ramp and is, therefore, treated as a post-cured reference state.

Figure 7 presents the absolute storage moduli $G^{\prime }\left(\omega \right)$ from isothermal DMA frequency sweeps. Panel (a) shows the four samples at 140 °C over the full 0.01–100 Hz window: $G^{\prime }$ rises monotonically with Shore A hardness, from ≈0.004 to 0.15 MPa for Pure EPDM through ≈0.008 to 0.38 MPa for EPDM60 and ≈0.018 to 0.58 MPa for EPDM70, to ≈0.26 to 2.0 MPa for EPDM80—more than an order of magnitude stiffer than the unfilled matrix at matched frequency. Panel (b) shows the temperature response of EPDM60: $G^{\prime }$ decreases between 120 and 140 °C (classical thermal softening), then rises at 160 °C (dashed). This non-monotonic behavior, together with the collapse of tanδ from ≳2 at 120 °C to ∼0.5 at 160 °C, is a known fingerprint of residual vulcanization during the ramp. We therefore treat the 160 °C isotherm as a post-cured reference state and restrict the quantitative frequency-slope analysis to 120 and 140 °C.

**Figure 8:**
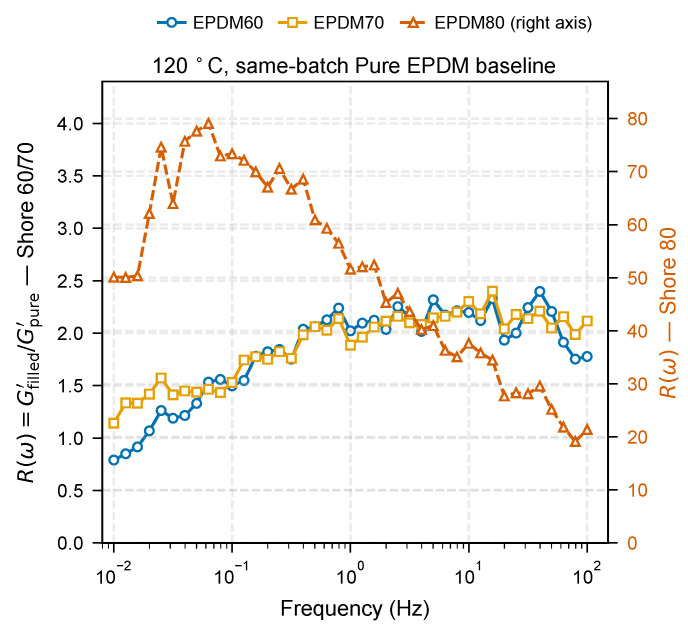
Frequency-resolved reinforcement factor $R\left(\omega \right)=G_{\mathrm{filled}}^{\prime }/G_{\mathrm{pure}}^{\prime }$ at 120 °C using the same-batch Pure EPDM as the reference matrix. EPDM60 and EPDM70 (Shore 60 and Shore 70) are read on the left axis; EPDM80 (Shore 80) is one order of magnitude higher and plotted on the right axis. The two lower-hardness grades show a mild frequency-dependent reinforcement increment above the low-frequency baseline, whereas EPDM80 sits in a filler-network-dominated regime with R(ω)≫10 across the entire frequency window.

The reinforcement factor $R\left(\omega \right)=G_{\mathrm{filled}}^{\prime }/G_{\mathrm{pure}}^{\prime }$ is shown in Figure 8. The denominator $G_{\mathrm{pure}}^{\prime }$ is the same-batch unfilled Pure EPDM measured under identical conditions, avoiding uncertainties from literature reference spectra. Focusing on the 120 °C isotherm, the Shore 60 and Shore 70 grades share a common reinforcement window with R(ω) rising from ∼1 at the lowest frequencies to ∼2–2.4 above 1 Hz, indicating a mild frequency-dependent increment above the low-frequency baseline. EPDM80 occupies a different reinforcement regime: its R(ω) reaches ∼50–80 with a weakly decreasing trend across the frequency window, reflecting the presence of a percolated filler network whose elastic contribution dominates over the soft unfilled matrix across the entire sweep. Because EPDM60/70 and EPDM80 span such different magnitudes, the latter is plotted on a secondary axis in Figure 8. Quantitative magnitude comparisons across the three Shore grades should be interpreted with caution because the compounds share the same carbon-black loading but differ in paraffinic oil content (60, 40, 20 phr for EPDM60, EPDM70, EPDM80; Section 2, Table 3), so the apparent reinforcement spectrum mixes intrinsic filler–polymer coupling with plasticizer-controlled matrix dilution. The analysis below therefore restricts causal claims to within-material temperature trends (frequency slope dR/dlogf of the same compound at 120 vs. 140 °C) rather than to absolute differences between compounds.

**Figure 9:**
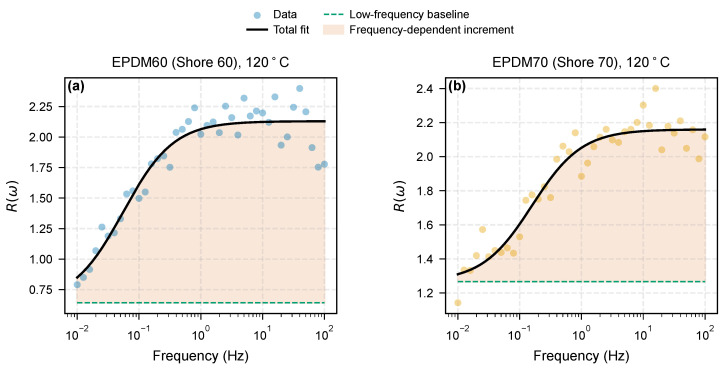
Phenomenological two-zone fits (Equation (2)) to the 120 °C reinforcement spectra for (**a**) EPDM60 and (**b**) EPDM70. The dashed green line marks the fitted low-frequency baseline, and the red shading indicates the frequency-dependent increment.

**Figure 10:**
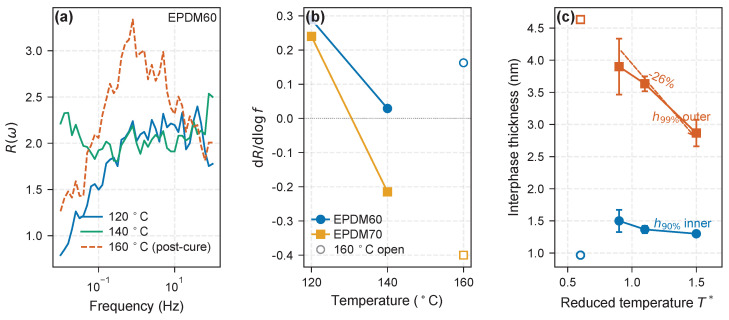
Temperature–frequency bridge between MD and DMA. (**a**) R(ω) spectra for EPDM60 at three temperatures; the 160 °C curve (dashed) is the post-cured reference. (**b**) Frequency slope dR/dlogf versus temperature for EPDM60 and EPDM70 (solid: 120/140 °C; open: 160 °C post-cured, excluded from fit). (**c**) MD flat-wall boundaries at $T^{*}=0.6$–1.5: $h_{90\%}$ contracts from 1.50 to 1.30 nm (13.3%), $h_{99\%}$ from 3.90 to 2.87 nm (26.5%). Open markers at $T^{*}=0.6$ denote the vitrification onset (large replica scatter, excluded from the contraction quantification); full statistics with Figure 3.

To decompose the frequency dependence, we fitted a phenomenological two-zone model:

(2) $$R\left(\omega \right)=R_{\mathrm{inner}}+R_{\mathrm{outer}}\;\frac{{\left(\omega /\omega_{c}\right)}^{n}}{1+{\left(\omega /\omega_{c}\right)}^{n}}$$

where $R_{\mathrm{inner}}$ represents the frequency-independent contribution of the densely adsorbed inner zone, $R_{\mathrm{outer}}$ captures the frequency-activated outer zone, and $\omega_{c}$ is the crossover frequency corresponding to the outer-zone relaxation time. Figure 9 shows the decomposition for the 120 °C spectra of EPDM60 and EPDM70. Within the measured window, the model reproduces the monotonic rise of R(ω) and cleanly separates a low-frequency baseline from a frequency-dependent increment. Table 5 reports the fitted parameters with the asymptotic ±1σ uncertainties from the covariance matrix of the nonlinear least-squares fit. For both materials the crossover frequency $\omega_{c}$ falls well below the accessible experimental window (0.06–0.16 Hz), so the inner- and outer-zone contributions are still partially correlated within the 0.01–100 Hz range and the individual parameters carry larger uncertainties than the total R(ω) envelope. The full Pearson correlation structure, derived from the same covariance matrix, is reported in Figure S3 of the Supplementary Materials; for both compounds, the $\left(R_{\mathrm{inner}},R_{\mathrm{outer}}\right)$ pair is anti-correlated at the −0.96 to −0.99 level, a direct consequence of the sub-window $\omega_{c}$. We therefore use the decomposition descriptively rather than as a unique inversion of inner- and outer-zone physics; the near-unity Hill exponent (n≈1.07 for both compounds) and the sign of the frequency slope (Figure 10) carry the physical interpretation.

**Table 5:** Phenomenological two-zone fit parameters for the 120 °C reinforcement spectra (Equation (2)), using same-batch Pure EPDM as the matrix reference. Uncertainties are asymptotic ±1σ values from the covariance matrix of the nonlinear least-squares fit. The crossover frequency $\omega_{c}$ falls below the experimental window for both compounds, producing residual correlation between $R_{\mathrm{inner}}$ and $R_{\mathrm{outer}}$; the fit is used descriptively.

Material	$R_{\mathrm{inner}}$	$R_{\mathrm{outer}}$	$\omega_{c}$ (Hz)	*n*	$R^{2}$
EPDM60	0.64±0.28	1.49±0.29	0.055±0.024	1.06±0.29	0.901
EPDM70	1.27±0.08	0.89±0.10	0.159±0.041	1.08±0.25	0.919

The selective thermal contraction of the outer zone (Section 3.2) predicts that dR/dlogf should diminish at higher temperatures. We test this using the 120 and 140 °C DMA isotherms of EPDM60 and EPDM70 (Figure 10b). The 160 °C response is shown on the same axes as open symbols for completeness but excluded from the quantitative fit because residual vulcanization at that temperature (Section 3.3, Figure 7b) makes the ratio cure-state-dependent rather than purely dynamical.

For EPDM60 the frequency slope decreases from dR/dlogf=+0.29 at 120 °C to +0.03 at 140 °C—a factor of ten reduction indicating that the outer-zone-mediated increment has essentially vanished by 140 °C. For EPDM70 the slope drops from +0.24 to −0.21 over the same interval, inverting sign between 120 and 140 °C: at 140 °C, higher-frequency motions no longer sample a stiffer interphase than lower-frequency motions. The 160 °C point is shown as open symbols in Figure 10b for completeness but is excluded from the thermal trend because it corresponds to a post-cured reference state (Section 3.3). Both trends at 120–140 °C are consistent with the predicted loss of the outer-zone frequency-dependent contribution on heating.

This experimental behavior parallels the MD finding that $h_{99\%}$ contracts by 26% between $T^{*}=0.9$ and 1.5 (3.90 → 2.87 nm), while $h_{90\%}$ contracts more weakly over the same range (13.3%; Figure 10c, Table 4). The three EPDM grades are industrial-style formulations in which Shore A hardness is set simultaneously by carbon-black and paraffinic oil content (60, 40, 20 phr for EPDM60/70/80 at fixed CB loading); this co-variation is a defining feature of the commercial grade system, not a processing artifact. Within a single grade the oil content is held constant, so the within-grade temperature response (e.g., the EPDM60 and EPDM70 dR/dlogf decrease from 120 to 140 °C above) is consistent with interphase-linked changes on heating, rather than strictly isolating an interphase contribution from matrix-mobility effects. A fixed-oil EPDM series would be required to separate the two (see limitations in Section 4). Across grades, R(ω) amplitudes combine interphase and plasticizer-mediated matrix-mobility contributions, as expected for industrial compounds, and are reported descriptively. Mapping zone thickness onto R(ω) amplitude quantitatively would require a micromechanical model beyond the scope of the present demonstration.

The simulated interphase thicknesses ($h_{99\%}\approx 2.8$–3.7 nm) are consistent with the 2–5 nm range reported by Berriot et al. [6,13] from NMR relaxation analysis and the 1.8–2.2 nm inferred by Salatto et al. [27] from neutron scattering and spin-echo measurements on polybutadiene/CB nanocomposites, with comparable few-manometer bound layers obtained by Bhutia et al. [28] via Guiselin-style swelling-kinetics analysis of annealed polymer thin films, suggesting that experimental techniques primarily probe the outer, loosely tethered zone rather than the thin inner adsorbed layer and supporting the bimodal picture on a plausible length scale.

### 3.4. Sensitivity to Chain Length and Surface Interaction

The interphase architecture depends on chain length and filler–polymer interaction strength, providing two additional handles beyond temperature. The MSD process curves (Figure 6a) show chain-length-dependent separation at matched loading, with the N=50 and 100 curves remaining above the N=25 curve over most of the sampled time window. Because the raw MSD ordering between N=50 and 100 is not strictly monotonic, the crossover discussion below is anchored by $h_{\mathrm{excess}}$, with $\alpha_{\mathrm{MSD},\mathrm{ens}}$ retained as a secondary dynamical indicator.

For N=25, $h_{\mathrm{excess}}$ remains between 0.11 and 0.40 nm from ϕ=5% to 25%, then jumps to 0.87 nm at ϕ=30% as adjacent interphase shells begin to overlap. For N=100, the interphase thickens much earlier—at ϕ=10%, $h_{\mathrm{excess}}=0.91$ nm compared with only 0.11 nm for N=25 (Table 2)—indicating that long chains bridge multiple fillers through loop and bridge conformations [16,17,29,30]. An ensemble check on every chain in the ϕ=10% boxes confirms this as a whole-box statistic, not an artifact of the three chains selected for Figure 5b–d: at a near-surface-contact cutoff $d_{\mathrm{cut}}=0.5$ nm the fraction of chains touching two or more distinct fillers rises monotonically from 41% (N=25) to 59% (N=50) to 81% (N=100), with the mean number of fillers visited per chain climbing from 1.5 to 2.2 to 4.1 (Figure S4). The same ordering holds at $d_{\mathrm{cut}}=0.75$ and 1.0 nm, so the bridging onset is robust to the contact threshold. This eight-fold difference at matched loading indicates a bridging onset within the N=25–100 interval: adsorption-dominated behavior at N=25 gives way to connectivity-assisted behavior at N=100. With only three chain lengths sampled at this loading we do not place the transition at a specific *N*.

Figure 5 plots $h_{\mathrm{excess}}$ versus ϕ for all three chain lengths. The companion-study N=50 data fall between the N=25 and 100 curves, consistent with a progressive crossover near N≈50 (Table 2). Both the N=25 and 100 series show non-monotonic scatter, reflecting the sensitivity of the excess density integral to the specific filler-packing realization in each ∼5000-bead simulation box: a single random placement of a small number of fillers controls whether neighboring interphase shells overlap, and the integrated excess is a volatile quantity at fixed ϕ until the box is large enough to average over many local packings. We do not fit a power law $h_{\mathrm{excess}}\left(\phi \right)$ within any single *N*; the mechanistic claim is restricted to the matched-loading contrast at ϕ=10%—where $h_{\mathrm{excess}}$ increases from 0.11 nm at N=25 to 0.91 nm at N=100—a factor of ∼8 that sits far above the point-to-point scatter within either series and is robust to any single outlying loading.

The ensemble MSD exponent $\alpha_{\mathrm{MSD},\mathrm{ens}}$ is treated as a secondary indicator because the log–log fit is noisier than the density-based metric and not uniformly well-conditioned across the ϕ-series. At ϕ=10% the short-chain system gives $\alpha_{\mathrm{MSD},\mathrm{ens}}=0.18$, close to the nanocomposite ensemble baseline ($\alpha_{\mathrm{MSD},\mathrm{ens}}=0.183$), whereas N=100 yields $\alpha_{\mathrm{MSD},\mathrm{ens}}=0.34$ at $h_{\mathrm{excess}}=0.91$ nm. These values appear alongside $h_{\mathrm{excess}}$ in Table 2; because the short-chain log–log fit is visibly weaker than its long-chain counterpart, we anchor the crossover to the density-based metric and use $\alpha_{\mathrm{MSD},\mathrm{ens}}\left(\phi \right)$ only for consistency checks.

### 3.5. Positioning Within Existing Interphase Models

The classical bound rubber theory of Berriot et al. [6,13] posits that filler particles are surrounded by a glassy shell whose thickness (∼2–5 nm) is controlled by the polymer segmental dynamics modified by confinement. Their DSC, DMA, and NMR measurements on poly(ethyl acrylate)/silica nanocomposites revealed a single immobilized layer with properties distinct from the bulk, but did not resolve internal stratification within that layer. Our simulations support the existence of such a modified layer, but further indicate a modest mobility stratification within it: the 1–2 nm outer zone has $\alpha_{\mathrm{MSD},\mathrm{layer}}\approx 0.495$, the >5.25 nm bulk reference reaches $\alpha_{\mathrm{MSD},\mathrm{layer}}\approx 0.527$, and the innermost 0–1 nm layer returns the (static-assignment) apparent value $\alpha_{\mathrm{MSD},\mathrm{layer}}\approx 0.55$ (see Section 3.2 and Supplementary Materials Table S3).

Phase-field approaches provide a complementary continuum description. Ginzburg [31] formulated a density-functional model for polymer–platelet nanocomposites that predicts interphase thickness scaling with filler loading and particle geometry; Zeng et al. [32] extended related ideas to clay nanocomposites, linking polymer–filler interaction parameters to macroscopic mechanical response. These continuum frameworks capture the overall interphase width but typically assume monotonic decay of the order parameter from the surface into the bulk. The bimodal density profiles observed in our MD simulations (Figure 1a) suggest that a simple gradient approximation may miss the plateau region at intermediate densities (0.5≲r≲1.5 nm), where the density remains elevated above the bulk value but far below the inner-zone peak. A two-step or double-well free energy functional might be required to capture this behavior.

Neutron scattering and MD analysis by Salatto et al. [27] identified structurally and dynamically distinct bound and mobile chain populations in polybutadiene/CB nanocomposites. While conceptually similar to our inner/outer zone distinction, their binary classification collapses the continuous spatial gradient into a single threshold. Our two-threshold analysis ($h_{90\%}$, $h_{99\%}$) indicates that a gradual transition, not a sharp discontinuity, better represents the interphase architecture, consistent with the smeared-out density profiles expected from polymer loop and bridge conformations. More recent CG simulations by Cheng et al. [33] and Betancourt et al. [34] have similarly resolved spatially heterogeneous dynamics in confined polymer films near surfaces, lending independent support to the two-zone mobility picture.

Table 6 summarizes the key differences between our bimodal framework and existing models.

**Table 6:** Comparison of theoretical frameworks for the polymer–filler interphase.

Model	Zone Structure	Temp. Dependence	Key Parameters
Berriot glassy shell [13]	Single layer	$T_{g}$ shift	Thickness, $\Delta T_{g}$
Ginzburg model [31]	Continuous gradient	Weak/implicit	Interface width, χ
Salatto NSE + MD [27]	Two populations	Not addressed	Dynamical persistence
**This work**	**Two zones**	**Outer zone thermally labile**	$h_{90\%,}$ $h_{99\%,}$$\alpha_{\mathrm{MSD},\mathrm{layer}}/\alpha_{\mathrm{MSD},\mathrm{ens}}$

The distinguishing feature of the present framework is the coupling of spatial stratification (inner vs. outer) with selective thermal activation of the outer zone. This asymmetry is absent from single-layer glassy shell models and not naturally captured by continuous phase-field approaches. It offers a molecular-scale account of the temperature-dependent frequency response in filled-elastomer DMA (Figure 10): on heating, outer-zone contraction erodes the frequency-dependent reinforcement increment while the inner-zone baseline responds more weakly.

Three routes modulate reinforcement through the bimodal interphase: (i) temperature contracts the outer zone more strongly than the inner zone (Section 3.2), with a corresponding loss of the frequency-dependent R(ω) increment at elevated temperatures (Figure 10); (ii) chain length *N* determines whether the interphase grows through local adsorption or non-local connectivity (Section 3.4); and (iii) surface chemistry ($\varepsilon_{\mathrm{wall}}$) modulates the adsorbed layer thickness and density (Figure 4), consistent with the enthalpic origin of the bound layer [5]. These levers are coupled: longer chains derive a larger share of their interphase from the outer zone, making their reinforcement more temperature- and frequency-sensitive.

### 3.6. Scope, Caveats, and Independent Validation

The paper’s central claim is the contrast between the two zones rather than the absolute precision of any single zone property. We therefore consolidate the principal caveats here so the quantitative envelope of every later inference is unambiguous.

*Replica budget for the temperature series*. Each flat-wall isotherm uses three independent NVT replicas. The headline contrast is the paired difference between $h_{99\%}$ and $h_{90\%}$ contractions (+13.2 pp, bootstrap 95% confidence interval [+7.5,+18.3], permutation p≈0.05). The contraction *ratio* (outer about twice inner) is the load-bearing claim and is robust because both percentiles are computed from the same trajectory, removing replica-level realization noise. Interpreting the inner- and outer-zone contractions as separately precise quantities would exceed what three replicas can support.

*Magnitude of the dynamic gradient*. The per-layer sub-diffusive exponent differs by $\Delta \alpha_{\mathrm{MSD}}\approx 0.03$ (≈6% of the bulk value) between the 1–2 nm outer zone and the bulk. The text labels this gradient *modest*, not strong. The structural evidence (bimodal density profile, paired contraction asymmetry, $\varepsilon_{\mathrm{wall}}$ sensitivity in Figure 4) carries the principal claim; the per-layer MSD acts as a corroborating indicator that the outer zone is not indistinguishable from bulk, not as the standalone justification for the two-zone picture.

*Chain-length sampling*. Only three lengths (N=25,50,100) are simulated. We deliberately do not assign a critical adsorption-to-bound transition length: the design instead brackets the unentangled (N=25) and entangled (N=100) regimes at matched filler loading, and reports the N=50 companion as an intermediate point. A finer-grid chain-length sweep is left to a follow-up study.

*Plasticizer covariation in the EPDM grades*. The EPDM60/70/80 series varies hardness by adjusting paraffinic oil content at fixed carbon-black loading. As discussed in Section 3.3, this is a defining feature of the industrial grade system rather than a processing artifact. Cross-grade R(ω) amplitudes therefore combine an interphase contribution with a plasticizer-mediated matrix-mobility contribution and are reported descriptively. The within-grade temperature response (e.g., the EPDM60 and EPDM70 dR/dlogf decrease from 120 to 140 °C) is at constant oil content and, therefore, consistent with interphase-linked thermal contraction. A fixed-oil EPDM series is the natural follow-up to isolate the interphase contribution from matrix softening.

*Boundary of the model in the temperature sweep*. The 160 °C isotherm sits at or beyond the upper end of the rubbery plateau accessed by these compounds and is reported as the post-cured reference (open markers in Figure 10b). It is intentionally excluded from the within-model frequency-slope fit. Within the calibrated 120–140 °C window the trend in dR/dlogf is monotonic and consistent with the two-zone picture; the model is not claimed to extrapolate beyond this window.

*Independent validation against published experimental DMA*. The viscoelastic envelope of the same EPDM formulation family has been independently characterized in a peer-reviewed companion study [19], in which the fixed-volume cumulative excess-density approach reproduces the DMA-anchored single-thickness $h_{\mathrm{bound}}$ values to within the reported uncertainty bands (Figure 1c and Figure 5d, open diamonds). This published comparator gives the reinforcement claims of the present work an independent experimental anchor that does not rely on the in-house ϕ-series simulations alone.

*Qualitative nature of the MD–DMA temperature bridge*. The reduced temperature $T^{*}$ used in the simulations is defined as $k_{B}T/\varepsilon$ within the Kremer–Grest framework and is not directly calibrated to EPDM thermal properties. The correspondence between the simulation window $T^{*}=0.9$–1.5 and the experimental window 120–140 °C is therefore qualitative: both windows lie well within the rubbery plateau of their respective systems. EPDM has $T_{g}\approx -52$ °C (221 K), placing 120–140 °C at $T/T_{g}\approx 1.87$–1.96; in the model, large replica scatter at $T^{*}=0.6$ (Table S1a) marks the onset of vitrification and sets an analogous lower bound, so the production window $T^{*}=0.9$–1.5 is similarly well above the model glass transition. The bridge claim is directional: the outer-zone boundary contracts roughly twice as much as the inner-zone boundary on heating in both simulation and experiment. Absolute thicknesses and temperatures are not claimed to be quantitatively calibrated, consistent with the approximate-conversion statement at the end of Section 2.

*Robustness of the bimodal topology to wall-potential choice*. The bimodal density stratification—a dense inner zone ($h_{90\%}$) surrounded by a broader outer zone ($h_{99\%}$)—is present in both wall-potential setups used in this work. The 12–6 Lennard–Jones confined-melt reference (*nanocomp*, $\varepsilon_{\mathrm{wall}}=1.0\;k_{B}T$) yields stratification ratio $h_{99\%}/h_{90\%}=8.3$, confirming the bimodal topology at the weakest physically reasonable wall interaction. The 9–3 Steele-type setup ($\varepsilon_{\mathrm{wall}}=2$–$8\;k_{B}T$) shows that stronger wall attraction alters the quantitative zone widths but preserves the two-zone structure across the full interaction-strength range (Figure 4). The bimodal topology arises from the competition between surface adsorption (which localizes a thin dense inner zone) and chain connectivity (which sustains a broader outer zone); it is not an artifact of a specific potential form.

## 4. Conclusions

Coarse-grained MD of flat-wall reference systems, cross-checked against EPDM/CB DMA spectra, resolves a polymer–filler interphase better described by two structurally and thermally distinct sublayers than by a homogeneous shell. The principal numerical findings, paired with their physical interpretation, are summarized below.

1. **Bimodal architecture with thermal selectivity.** The two zone boundaries respond selectively on heating: the inner-zone $h_{90\%}$ contracts by 13.3%, while the outer-zone $h_{99\%}$ contracts by 26.5%, giving a paired contrast of +13.2 percentage points (bootstrap 95% confidence interval [+7.5,+18.3]). The per-layer MSD exponent differs by $\Delta \alpha_{\mathrm{MSD}}\approx 0.03$ between the 1–2 nm outer zone and the bulk, a modest mobility gradient consistent with the stratification. Models that truncate the bound layer at $h_{90\%}$ plausibly underestimate the dynamically active volume.
2. **Chain-length-dependent transition.** At ϕ=10vol%, the inter-filler bridging fraction rises from 41% at N=25 to 81% at N=100, while the cumulative excess thickness $h_{\mathrm{excess}}$ grows from 0.11 to 0.91 nm over the same range. The transition is therefore bracketed by the N=25–100 window rather than assigned to a specific critical chain length.
3. **Three coupled design levers.** Filler loading (ϕ), surface interaction strength ($\varepsilon_{\mathrm{wall}}$), and chain length (*N*) jointly reshape the bimodal interphase, providing a molecular rationale for the temperature–frequency coupling observed in filled-elastomer DMA master curves.
4. **Scope and outlook.** The replica budget for the temperature series, the $\Delta \alpha_{\mathrm{MSD}}$ magnitude, the chain-length grid, the plasticizer covariation in the EPDM60/70/80 series, and the 160 °C model boundary are discussed in Section 3.6 together with the independent experimental validation against a peer-reviewed companion DMA study [19]. Larger boxes, additional replicas, a fixed-oil EPDM series, a nanocomposite temperature series, and physics-based links from the density profile to the dynamic modulus are the natural follow-ups.

## Data Availability

Processed data underlying the main text and Supplementary Materials, together with selected simulation outputs and representative LAMMPS input decks for the flat-wall and nanocomposite simulations, are provided in the review data archive submitted with the manuscript. Analysis and plotting scripts, raw LAMMPS trajectories, and raw DMA source files are available from the corresponding author on reasonable request.

## Supplementary Materials

The following supporting information can be downloaded at: https://www.mdpi.com/article/10.3390/molecules31101615/s1, Figures S1–S4 (threshold sensitivity; direct radial density profile; two-zone parameter correlation; ensemble bridging fraction) and Tables S1–S3 (per-replica $h_{90\%}$/$h_{99\%}$ with robust statistics; LAMMPS velocity seeds; block-bootstrap sensitivity of the per-layer MSD exponent). Processed data files, selected simulation outputs, and representative LAMMPS input decks are available as stated in the Data Availability Statement.

## Abbreviations

The following abbreviations are used in this manuscript:

CG-MD	Coarse-grained molecular dynamics
DMA	Dynamic mechanical analysis
EPDM	Ethylene–propylene–diene monomer (terpolymer rubber)
CB	Carbon black
LJ	Lennard–Jones (potential)
NPT, NVT, NVE	Constant-(N,P,T), constant-(N,V,T), constant-(N,V,E) statistical ensembles
MSD	Mean-squared displacement
$\alpha_{\mathrm{MSD}}$	Sub-diffusive log–log slope of MSD vs. *t*
$h_{90\%},h_{99\%}$	Inner-/outer-zone interphase boundaries (density-recovery thresholds)
$h_{\mathrm{excess}}$	Cumulative excess-density integral (nanocomposite geometry)
$h_{\mathrm{bound}}$	Single-thickness “bound rubber” descriptor used in classical models
R(ω)	Frequency-dependent reinforcement factor $G_{\mathrm{filled}}^{\prime }/G_{\mathrm{matrix}}^{\prime }$
LVR	Linear viscoelastic region
NSE	Neutron spin-echo (literature reference technique)
NMR	Nuclear magnetic resonance (literature reference technique)
DSC	Differential scanning calorimetry (literature reference technique)
phr	Parts per hundred rubber (industrial loading unit)
