# Supplementary material for: Bimodal Interphase Architecture in Filled Elastomers: Molecular Dynamics Evidence and Experimental Signatures
Source: Molecules. 2026 May 11;31(10):1615. doi: 10.3390/molecules31101615 (PMC13209950; doi:10.3390/molecules31101615)
Supplement: Supplementary file 1 [file molecules-31-01615-s001.zip › molecules-4299852-supplementary.pdf]

Abstract

This file p rovides t he s upplementary m aterials f or t he m ain m anuscript, including threshold-sensitivity analysis, direct radial-density evidence, parameter-correlation diagnostics, ensemble bridging statistics, and supplementary tables for robust statistics and reproducibility.

Keywords: supplementary materials; bound rubber; interphase heterogeneity; molecular dynamics; filled elastomers

This document contains four supplementary figures (Figures S1–S4) and three supplementary tables (Tables S1–S3) referenced in the main article. Processed data files, selected simulation outputs, and representative LAMMPS input decks that underlie the figures and tables in the main text and Supplementary Materials are consolidated in the review data archive submitted with the manuscript for peer review. A public Zenodo DOI will be provided at acceptance. Analysis and plotting scripts, raw LAMMPS trajectories, and raw DMA source files are available from the corresponding author on reasonable request.

S1. Threshold sensitivity of the outer-zone contraction

To verify that the $T^* = 0.9 \to 1.5$ outer-zone contraction is not an artefact of the density-threshold convention, Figure S1 reports h_p for $p = 0.85$–0.995 and three independent density-profile binnings (0.05 œ, 0.10 œ, 0.20 œ). The contraction is positive at every threshold and for every binning choice.

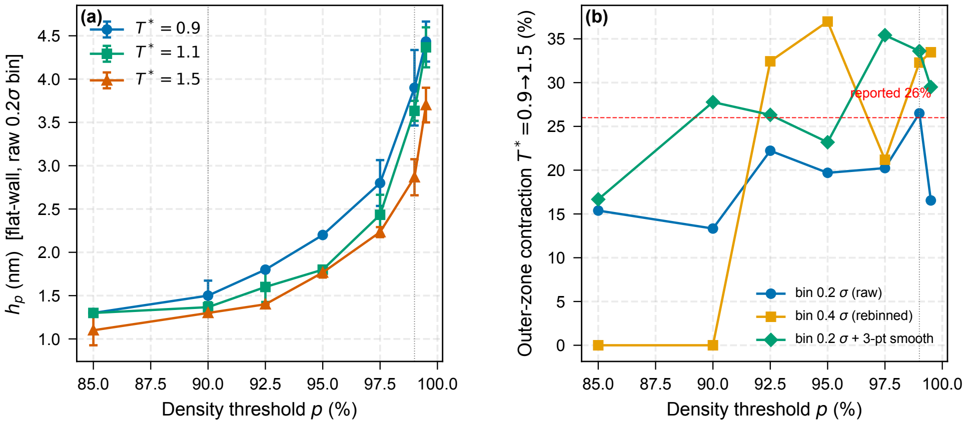

Figure S1. Threshold-sensitivity analysis. (a) h_p at $T^* = 0.9$ and $T^* = 1.5$ for $p \in [0.85, 0.995]$; bands denote replica spread ($n = 3$). (b) $T^* = 0.9 \to 1.5$ contraction (%) vs. p. The reported $h_{99\%}$ contraction (26.5%, CI $[17.4, 34.8]$%) lies within the shaded envelope. Source: `data/md_hbound/SI_threshold_sensitivity.csv`, `SI_threshold_contraction.csv`.

S2. Direct nanocomposite radial density profile

Because the curved-filler geometry smears the two zones into an overlapping radial profile that we cannot independently decompose in the main text, Figure S2 shows the raw radial density $\rho(r)/\rho_{\text{bulk}}$ measured from the filler surface for the $N = 50$, $\phi = 10$, 20 and 30% nanocomposites, together with two quantiles of the radial cumulative excess integral, $h^{\text{cum}}_{90\%}$ and $h^{\text{cum}}_{99\%}$, defined as the distances at which the running integral $\int_0^r \max[\rho(r')/\rho_{\text{bulk}} - 1, 0]\, dr'$ reaches 90% and 99% of its total. These are *cumulative-excess quantiles* and differ operationally from the density-threshold first-crossing h_p used in the flat-wall analysis of the main text (Section 2.3); the two conventions are presented side by side to show that a two-tier structure is detectable in the direct nanocomposite geometry, not to quote identical numbers.

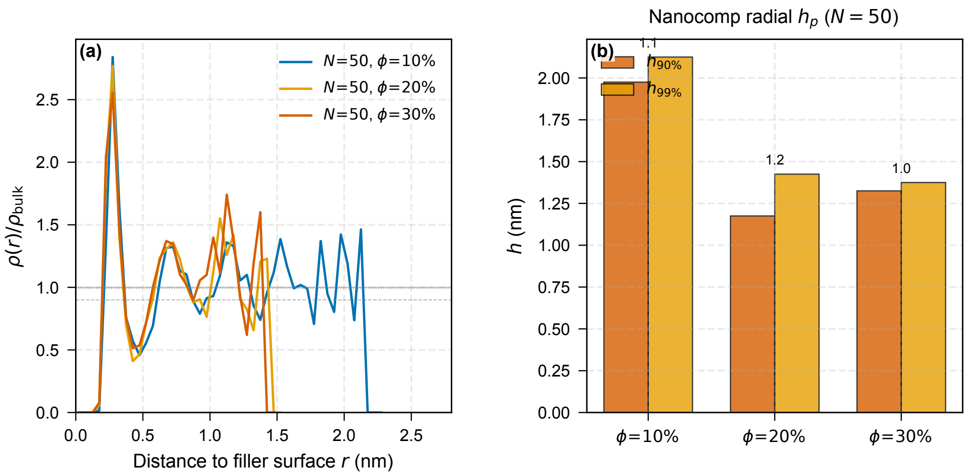

Figure S2. Nanocomposite radial density profile and the two cumulative-excess quantiles $h_{90\%}^{\mathrm{cum}}$ and $h_{99\%}^{\mathrm{cum}}$ at three filler loadings for $N = 50$, $T^* = 1.0$. The quantities are the radial distances at which the running integral of $\max[\rho/\rho_{\mathrm{bulk}} - 1, 0]$ reaches 90% and 99% of its total; they are distinct from the density-threshold first-crossing h_p used in the flat-wall analysis of the main text. At $\phi = 10\%$ the two quantiles are close together ($\approx$ 1.1 and 1.2 nm), reflecting that the outer-zone plateau seen in the flat wall is largely suppressed in the curved-filler geometry where neighbouring interphase shells overlap. Source: `data/md_hbound/SI_nanocomp_radial.csv`.

S3. Parameter correlation of the two-zone fit

The four-parameter two-zone model $R(\omega) = R_{\mathrm{inner}} + R_{\mathrm{outer}}\,(\omega/\omega_c)^n/[1 + (\omega/\omega_c)^n]$ fitted to the DMA reinforcement spectra (main-text Equation 1, Table 4) can exhibit parameter trade-offs when the transition frequency ω_c drifts outside the measured window. Figure S3 reports the Pearson correlation matrix of $(R_{\mathrm{inner}}, R_{\mathrm{outer}}, \omega_c, n)$ derived from the covariance matrix of the nonlinear least-squares fit.

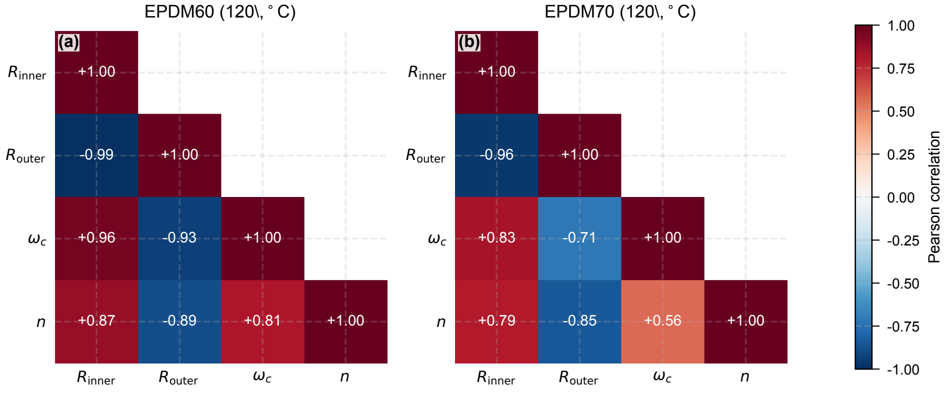

Figure S3. Parameter correlation matrices for (a) EPDM60 and (b) EPDM70. The strong anti-correlation between R_{inner} and R_{outer} ($|r| > 0.9$ for EPDM70) arises when ω_c falls below the measured window and the outer-zone amplitude compensates for an over-estimated inner-zone baseline. This trade-off is reported as a caveat to the main-text Table 5 ω_c values.

S4. Ensemble bridging fraction vs. chain length

Figure S4 complements main-text Figure 5b–d (which renders the three chains of largest spatial extent at each N) with the ensemble statistic over *every* polymer chain in the $\phi = 10\%$, $T^* = 1.0$ boxes, at three near-surface-contact cutoffs ($d_{\text{cut}} = 0.5,\ 0.75,\ 1.0$ nm; $d_{\text{cut}} = 0.5$ nm is the primary value).

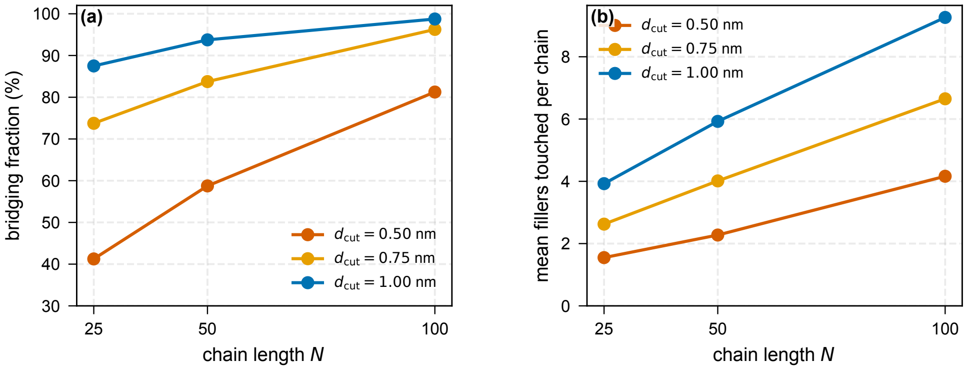

Figure S4. (a) Fraction of chains touching ≥ 2 distinct fillers as a function of chain length N. At the primary 0.5 nm cutoff the bridging fraction rises monotonically from 41% ($N = 25$) to 81% ($N = 100$); the monotonic trend persists at all three cutoffs, demonstrating that the adsorption-to-bridging transition is not a cutoff artefact. (b) Mean number of distinct fillers visited per chain. Source: `data/md_hbound/SI_bridging_fraction.csv`.

S5. Robust statistics for the outer-zone contraction (Table S1)

Table S1 lists (panel a) the per-replica $h_{90\%}$ and $h_{99\%}$ values used in the main-text robust-statistics analysis and (panel b) the aggregate robust statistics (mean, SD, bootstrap 95% CI of the $0.9 \to 1.5$ contraction, Cohen's d, and the exact-permutation p-value). The permutation test enumerates all $\binom{6}{3} = 20$ partitions of the six replicas into two groups of three and reports the fraction with mean difference $\geq$ observed; with $n = 3 + 3$ the minimum achievable p is $1/20 = 0.050$, which is the value obtained for both quantities. Panel c reports the *paired contrast* $\Delta h_{99\%} - \Delta h_{90\%}$ (Phase 2 addition, requested by the reviewer) to directly test whether the outer-zone contraction is significantly larger than the inner-zone contraction.

Table S1. Robust statistics for the $T^* = 0.9 \to 1.5$ outer-zone contraction: (a) per-replica thicknesses; (b) aggregate statistics (CI from a 10^4-resample nonparametric bootstrap; Cohen's d from pooled SD; exact permutation p from the $\binom{6}{3} = 20$ partitions, one-sided, $\geq$-observed); (c) paired contrast between outer and inner contractions.

(a) Per-replica thicknesses ($n = 3$ at each T^*).

T^*	replica	$h_{90\%}$ (nm)	$h_{99\%}$ (nm)
0.9	r1	1.4	3.7
0.9	r2	1.7	4.4
0.9	r3	1.4	3.6
1.5	r1	1.3	2.8
1.5	r2	1.3	2.7
1.5	r3	1.3	3.1

(b) Aggregate contraction statistics.

Quantity	$\langle h \rangle_{T^*=0.9}$ (nm)	$\langle h \rangle_{T^*=1.5}$ (nm)	Contraction (%)	95% CI (%)	Cohen's d	p_{perm}
$h_{99\%}$ (outer zone)	3.90	2.87	26.5	[17.4, 34.8]	3.03	0.050
$h_{90\%}$ (inner zone)	1.50	1.30	13.3	[7.1, 23.5]	1.63	0.050

(c) Paired contrast: $\Delta_{99} - \Delta_{90}$ where $\Delta_p = (\langle h_p \rangle_{0.9} - \langle h_p \rangle_{1.5}) / \langle h_p \rangle_{0.9}$.

Statistic	Value	95% CI	p_{boot}	p_{perm}
$\Delta_{99} - \Delta_{90}$ (pp)	+13.2	[+7.5, +18.3]	$< 10^{-3}$	0.050

Paired bootstrap (B=10^4, seed 42) resamples replica tuples $(h_{99\%}, h_{90\%})$ within each T^*, preserving the within-replica correlation between the two percentiles. Exact paired permutation enumerates all $\binom{6}{3} = 20$ partitions of the six *pairs* and recomputes the contrast statistic; $p_{\text{perm}} = 1/20 = 0.050$ is the floor attainable with $n = 3 + 3$. The combined evidence—effect sizes $d = 3.03$ vs. $d = 1.63$ and a paired contrast whose bootstrap CI excludes zero—separates the two zones well beyond the $p = 0.050$ floor of the group-wise test. Source: `data/md_hbound/SI_temperature_{robust_replicas,robust_stats,paired_contrast}.csv`.

S6. LAMMPS velocity seeds (Table S2)

For full reproducibility of the flat-wall temperature-series statistics we document the random-number seeds used to generate the initial velocity distribution of each of the 12 replicas. Each seed is applied via an explicit `velocity all create 1.0 <seed>` statement in the corresponding `flatwall_T*_r*.log`; the header-level `variable SEED index 54321` is a placeholder only. All runs use a single NVT integrator, so no separate Langevin seed is required.

Table S2. LAMMPS velocity seeds for the 12 flat-wall temperature-series replicas (4 isotherms × 3 replicas).

T^*	**r1**	**r2**	**r3**
0.6	60 321	75 838	83 757
0.9	63 321	105 838	113 757
1.1	65 321	125 838	133 757
1.5	69 321	165 838	173 757

S7. Block-bootstrap sensitivity of per-layer α (Table S3)

The per-layer MSD exponents α_ℓ shown in main-text Figure 2(b) carry uncertainties estimated by a moving-block bootstrap that preserves serial correlation of the MSD trajectory ($B = 2000$ resamples, block size $= 25$ time points, log–log window $100 \leq t \leq 2500\,\tau$). Table S3 scans block sizes $\{15, 20, 25, 30, 35\}$ to verify that the reported σ_α is not an artefact of the chosen block length.

Table S3. Per-layer $\langle\alpha\rangle$ (bootstrap mean) and σ_α (bootstrap SD of the log–log slope) as a function of block size. Rows are interphase and bulk layers; columns give $(\langle\alpha\rangle, \sigma_\alpha)$ at five block sizes. The sensitivity of σ_α is $\leq$ 0.006 for interphase layers and $\leq$ 0.013 for the bulk layer, well below the outer-zone–to–bulk α-difference ($\approx$ 0.032) reported in the main text. The $\langle\alpha\rangle$ values at the default block size 25 are the canonical per-layer exponents quoted in the main text and plotted in main-text Figure 2b—both computed from the same block-bootstrap definition (no separate JSON layer summary is used). Source: `data/md_hbound/SI_block_sensitivity.csv` and `data/md_hbound/per_layer_alpha_canonical.csv`.

Layer	bs=15<br>α / σ	bs=20<br>α / σ	bs=25<br>α / σ	bs=30<br>α / σ	bs=35<br>α / σ
0–1 nm	0.553 / 0.008	0.553 / 0.010	0.553 / 0.011	0.553 / 0.013	0.557 / 0.014
1–2 nm	0.496 / 0.007	0.496 / 0.008	0.495 / 0.009	0.495 / 0.009	0.497 / 0.012
2–3.5 nm	0.487 / 0.006	0.487 / 0.007	0.487 / 0.008	0.488 / 0.008	0.487 / 0.010
3.5–5.25 nm	0.509 / 0.012	0.509 / 0.013	0.508 / 0.016	0.510 / 0.016	0.507 / 0.015
$>$5.25 nm	0.528 / 0.018	0.529 / 0.022	0.527 / 0.026	0.531 / 0.030	0.523 / 0.030
